# Enhanced characteristics of genetically modified switchgrass (*Panicum virgatum* L.) for high biofuel production

**DOI:** 10.1186/1754-6834-6-71

**Published:** 2013-05-07

**Authors:** Hui Shen, Charleson R Poovaiah, Angela Ziebell, Timothy J Tschaplinski, Sivakumar Pattathil, Erica Gjersing, Nancy L Engle, Rui Katahira, Yunqiao Pu, Robert Sykes, Fang Chen, Arthur J Ragauskas, Jonathan R Mielenz, Michael G Hahn, Mark Davis, C Neal Stewart, Richard A Dixon

**Affiliations:** 1 Plant Biology Division, Samuel Roberts Noble Foundation, 2510 Sam Noble Parkway, Ardmore, OK, 73401, USA; 2 Department of Plant Sciences, University of Tennessee, 2431 Joe Johnson Dr., Knoxville, TN, 37996, USA; 3 National Renewable Energy Laboratory, 1617 Cole Blvd., Golden, CO, 80401, USA; 4 Complex Carbohydrate Research Center, University of Georgia, 315 Riverbend Rd., Athens, GA, 30602, USA; 5 School of Chemistry & Biochemistry, Georgia Institute of Technology, 901 Atlantic Drive, 30332, Atlanta, GA, USA; 6 BioEnergy Science Center (BESC), Oak Ridge National Laboratory, Oak Ridge, TN, 37831, USA; 7 Present address: Department of Biological Sciences, University of North Texas, 1155 Union Circle, Denton, TX 76203, USA

**Keywords:** Switchgrass, Bioenergy, Biofuel, Feedstock, Cellulosic ethanol, PvMYB4, Transcription factor, Cell wall, Recalcitrance, Lignin, Hemicellulose, Pectin

## Abstract

**Background:**

Lignocellulosic biomass is one of the most promising renewable and clean energy resources to reduce greenhouse gas emissions and dependence on fossil fuels. However, the resistance to accessibility of sugars embedded in plant cell walls (so-called recalcitrance) is a major barrier to economically viable cellulosic ethanol production. A recent report from the US National Academy of Sciences indicated that, “absent technological breakthroughs”, it was unlikely that the US would meet the congressionally mandated renewable fuel standard of 35 billion gallons of ethanol-equivalent biofuels plus 1 billion gallons of biodiesel by 2022. We here describe the properties of switchgrass (*Panicum virgatum*) biomass that has been genetically engineered to increase the cellulosic ethanol yield by more than 2-fold.

**Results:**

We have increased the cellulosic ethanol yield from switchgrass by 2.6-fold through overexpression of the transcription factor PvMYB4. This strategy reduces carbon deposition into lignin and phenolic fermentation inhibitors while maintaining the availability of potentially fermentable soluble sugars and pectic polysaccharides. Detailed biomass characterization analyses revealed that the levels and nature of phenolic acids embedded in the cell-wall, the lignin content and polymer size, lignin internal linkage levels, linkages between lignin and xylans/pectins, and levels of wall-bound fucose are all altered in PvMYB4-OX lines. Genetically engineered PvMYB4-OX switchgrass therefore provides a novel system for further understanding cell wall recalcitrance.

**Conclusions:**

Our results have demonstrated that overexpression of PvMYB4, a general transcriptional repressor of the phenylpropanoid/lignin biosynthesis pathway, can lead to very high yield ethanol production through dramatic reduction of recalcitrance. MYB4-OX switchgrass is an excellent model system for understanding recalcitrance, and provides new germplasm for developing switchgrass cultivars as biomass feedstocks for biofuel production.

## Background

Bioethanol from cellulosic feedstocks such as corn stover, switchgrass or wood chips, is a promising renewable and clean energy source, with the potential to reduce greenhouse gas emissions by up to 86% compared with gasoline [1]. However, ethanol production from lignocellulosic materials faces more challenges than from starch-based feedstocks as a result of the chemical and physical barriers that block accessibility to the sugars (so-called recalcitrance) within the biomass. Pretreatment is required to partially deconstruct the biomass and open up surfaces for enzymatic hydrolysis to release 5- and 6-carbon sugars for fermentation. Pretreatment is not only expensive [2], but also produces inhibitors of microbial ethanol fermentation such as 2-furaldehyde (furfural) and 5-hydroxymethylfurfural (HMF) during acidic pretreatments [3].

Switchgrass has attractive features as a dedicated lignocellulosic feedstock for bioenergy production in the United States [4-6], and recent studies report partial success in overcoming recalcitrance. For example, down-regulation of cinnamyl alcohol dehydrogenase (CAD), the last enzyme of lignin precursor formation, increases saccharification efficiency up to 23% without acid pretreatment [7,8]. Likewise, down-regulation of caffeic acid 3-*O*-methyltransferase (COMT), a key enzyme for biosynthesis of the monolignol sinapyl alcohol, increases saccharification efficiency by 29-38% without acid pretreatment [9]. However, reduction of sinapyl monolignol production may increase concentrations of fermentation inhibitors [10], and low molecular weight phenolic compounds in *COMT* down-regulated switchgrass inhibit simultaneous saccharification and fermentation (SSF) by the yeast *Saccharomyces cerevisiae* unless first removed by hot water pretreatment [11]. Clearly, a better strategy for reducing recalcitrance is required for the development of improved lignocellulosic bioenergy feedstocks.

Overexpression of the switchgrass R2-R3 MYB transcription factor *PvMYB4* in switchgrass represses lignin biosynthetic pathway genes and increases saccharification efficiency up to 300% without acid pretreatment [12]. Here, we evaluate the bioconversion of such materials to ethanol using yeast-based SSF methods. Metabolite profiling revealed major reductions in levels of phenolic fermentation inhibitors. Furthermore, application of a suite of chemical, immunological, and physical approaches for cell wall characterization revealed that multiple components, including lignin and wall-bound phenolics, pectin-lignin and xylan-lignin linkages, and fucosylated xyloglucans and rhamnogalacturonans, could potentially contribute to recalcitrance.

## Results and discussion

### PvMYB4 overexpression in switchgrass

Previously generated PvMYB4-over-expressing (PvMYB4-OX) transgenic switchgrass lines (1A, 1B, 1C, 1D, 1E, 2A and 2B) were in the Alamo ST2 genetic background [12], and additional lines were constructed in Alamo ST1 (Additional file 1: Figure S1a). Nine regenerated plants were selected from independent antibiotic resistant calli, and six lines (L1, L2, L4, L6, L8 and L11) were confirmed to be transgene positive by genomic DNA PCR (Additional file 1: Figure S1b). The PvMYB4 expression level was determined by qRT-PCR analysis (Additional file: 1 Figure S1c). Lines L6 and L8 showed intermediate expression level compared to lines L1, L2, L4 and L11. Overexpression of *PvMYB4* repressed endogenous *PvMYB4* expression, indicating a negative self-regulatory mechanism (Additional file 1: Figure S1d). Adult PvMYB4-OX plants showed reduced tiller height and tiller diameter, but increased tiller numbers in both genetic backgrounds under greenhouse conditions [12], Additional file 1: Figure S1e). Whole tillers (comprised of approximately 48% leaves and 52% stems on a weight basis for both control and transgenic materials) were used in all the following experiments as these represent the material that would be processed in a biorefinery. All materials were harvested at the same developmental stage (R1) according to a recently published protocol designed to facilitate comparisons between transgenic and control switchgrass materials [13].

### PvMYB4-OX lines exhibit up to a 2.6-fold increase in ethanol yield

**Figure 1:**
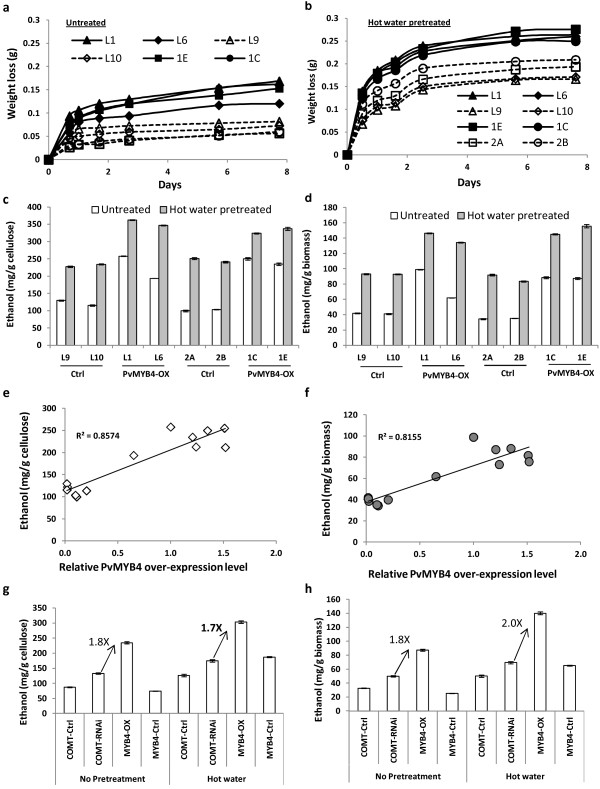
**Bioconversion of PvMYB4-OX transgenic switchgrass biomass to ethanol with or without hot water pretreatment using *Saccharomyces cerevisiae *D5A.** (**a**, **b**) Time courses of fermentation of whole plant material without (**a**) and with (**b**) hot water pre-treatment in fermentation broths measured by weight loss. (**c**, **d**) Final ethanol yields calculated as mg/g cellulose (**c**) or mg/g biomass (**d**) from hot water pre-treated and non-treated biomass, with comparison of different control (Ctrl) and PvMYB4-OX lines. (**e**, **f**) Correlation between the PvMYB4 transcript level and SSF ethanol yield without pretreatment. Data from lines L1, L6, L7, L8, L9 and L10 (Alamo ST1 background), and 1A, 1B, 1C, 1D, 1E, 2A and 2B) Alamo ST2 genetic background) are used. (**g**, **h**) SSF ethanol yield comparison of PvMYB4-OX and PvCOMT-RNAi [9] transgenic lines. All data are means ± SE (n = 3).

Ethanol yields of control and PvMYB4-OX switchgrass were first assessed by weight loss during yeast-based SSF with or without hot-water pretreatment (Figure 1a and 1b). PvMYB4-OX biomass underwent a faster hydrolysis of cellulose to glucose and faster conversion of the glucose to ethanol and CO_2_ under both pretreated and non-pretreated conditions than did control material. After 7 days fermentation, the ethanol yield per gram of cellulose or biomass was about 2.6-fold higher for the MYB4-OX lines than the control lines under non-pretreated conditions (Figure 1c, d). After hot water pretreatment, the ethanol yield increased significantly in both control and MYB4-OX lines. However, untreated MYB4-OX transgenic biomass had a similar ethanol yield to pretreated control biomass (Figure 1c, d). HPLC (High-performance liquid chromatography) analysis indicated that only 0.077 to 0.175 mg glucose per gram of dry biomass was left in the fermentation medium, and no furfural or HMF were detected. The SSF ethanol yield without pretreatment showed a strong positive correlation (R^2^ > 0.8) with the expression level of PvMYB4 (Figure 1e, f). PvMYB4-OX switchgrass produces approximately 1.8-fold more ethanol than COMT-RNAi switchgrass [9] under the same conditions (Figure 1g, h).

### PvMYB4-OX switchgrass has reduced levels of phenolic fermentation inhibitors

To check for the fermentation inhibitors reported in COMT-RNAi switchgrass [11], we performed metabolite profiling of methanol extractives using GC-MS (Table 1). Over 160 peaks were examined. Levels of phenolic fermentation inhibitors such as aromatic aldehydes (*p*-hydroxybenzaldehyde and coniferaldehyde) and organic acids (*p*-coumaric [*p*-CA], ferulic [FA] and sinapic acids) were significantly reduced in the PvMYB4-OX material (Table 1). Levels of various lignans were either reduced or increased in PvMYB4-OX lines (Table 1). The potential of lignans as fermentation inhibitors is unclear and needs further evaluation.

**Table 1:** Metabolite concentrations (ng/ml; sorbitol equivalents) of methanol extractives by GC-MS

**Metabolite**	**MYB4**-**OX**	**Control**	**MYB4**-**OX**/**Control**	***P***-**value**	**Classification**
16.07 375 583 513 411 427 204	6	1	8.66	0.000	glucoside
16.23 488 327 265 syringyl lignan	4	1	6.33	0.018	lignan
15.97 583 375 285	70	12	5.98	0.001	N/A
11.01 450 217 sugar	45	11	4.29	0.030	modified sugar
9.96 281 383 354	289	71	4.07	0.022	phenolic
10.27 328 343 284 254	999	245	4.08	0.005	N/A
raffinose	933	267	3.49	0.022	sugar - trisaccharide
galactose	1193	345	3.46	0.056	sugar - monosaccharide
19.47 496 481 209 lignan	36	11	3.31	0.050	lignan
α-tocopherol	18	6	3.20	0.060	vitamin
γ-tocopherol	8	3	2.73	0.018	vitamin
13.93 375 292 305 275 uronic acid	176	69	2.54	0.002	sugar acid conjugate
dehydroabietic acid	76	32	2.36	0.048	resin acid
19.09 483 498 lignan	11	5	2.27	0.047	lignan
tryptophan	264	120	2.21	0.036	amino acid
16.11 368 600 585 353 255	11	5	2.18	0.001	N/A
fructose	8160	3897	2.09	0.083	sugar - monosaccharide
bornesitol	2914	1403	2.08	0.000	cyclitol
glutamine	605	315	1.92	0.081	amino acid
tyrosine	234	123	1.91	0.058	amino acid
α-linolenic acid	1132	674	1.68	0.060	fatty acid
alanine	3061	1835	1.67	0.002	amino acid
glucose	6307	3799	1.66	0.040	sugar - monosaccharide
16.85 caffeic acid conjugate	17	11	1.59	0.031	phenylpropanoid
dodecanoic acid	134	88	1.53	0.090	fatty acid
3-*O*-caffeoylquinic acid	414	297	1.39	0.146	phenylpropanoid
5-*O*-caffeoylquinic acid	24	17	1.37	0.073	phenylpropanoid
caffeic acid	42	30	1.37	0.040	phenylpropanoid
GABA (γ-aminobutyric acid)	7451	5457	1.37	0.050	amino acid
shikimic acid	6025	4626	1.30	0.423	organic acid
campesterol	54	43	1.26	0.078	sterol
4-*O*-caffeoylquinic acid	28	23	1.23	0.405	phenylpropanoid
quinic acid	2716	2670	1.02	0.932	organic acid
sinapic acid	4	6	0.78	0.075	phenylpropanoid
5-*O*-feruloylquinic acid	134	185	0.72	0.050	phenylpropanoid
19.14 572 498 483	2	2	0.70	0.001	N/A
5-hydroxyconiferyl alcohol	1	1	0.68	0.001	phenylpropanoid
ferulic acid	26	42	0.61	0.001	phenylpropanoid
sinapyl alcohol	6	10	0.58	0.004	phenylpropanoid
sucrose	3600	6212	0.58	0.226	sugar - disaccharide
4-*O*-feruloylquinic acid	98	173	0.57	0.013	phenylpropanoid
12.88 553 463 373 283	27	49	0.56	0.001	N/A
coniferaldehyde	1	1	0.53	0.006	phenylpropanoid
*p*-coumaric acid	102	236	0.43	0.000	phenylpropanoid
3-*O*-feruloylquinic acid	171	413	0.41	0.007	phenylpropanoid
16.32 327 syringyl lignan	227	562	0.41	0.000	lignan
coniferyl alcohol	4	12	0.36	0.000	phenylpropanoid
16.11 327 297 syringyl lignan	23	66	0.35	0.002	lignan
16.76 354 482 439 323 297 lignan	1	3	0.32	0.000	lignan
syringin	3	10	0.31	0.000	phenylpropanoid
16.82 354 456 203 188	0	1	0.30	0.002	N/A
16.06 297 guaiacyl lignan	644	2184	0.30	0.000	lignan
9.99 275	1	4	0.20	0.000	N/A
*p*-hydroxybenzaldehyde	4	23	0.18	0.000	phenylpropanoid
guaiacylglycerol	45	287	0.16	0.000	phenylpropanoid
15.84 412 323 297 209 lignan	29	207	0.14	0.000	lignan
15.12 518 shikimic acid conjugate	0	2	0.13	0.001	organic acid
16.62 486 576 546 456 209 lignan	9	72	0.13	0.000	lignan

Unidentified and tentatively identified metabolites are designated by retention time (RT; min) and key mass-to-charge (m/z) ratios, with the first designated m/z extracted for relative quantification.

The content of soluble phenolics extracted by 50% methanol from the whole biomass of PvMYB4-OX lines was reduced by about 10-20% compared to controls (Additional file 1: Figure S2a). Levels of the monolignols coniferyl alcohol, sinapyl alcohol and its glucoside syringin, and 5-hydroxyconiferyl alcohol were all reduced. Levels of feruloylquinic acid esters declined, whereas levels of caffeoylquinic acid esters were unchanged. Levels of the soluble sugars glucose, fructose, galactose and raffinose were increased in the methanol extractives of MYB4-OX lines, by from 1.6- to 3.5-fold. These increases in monosaccharides, with sucrose unchanged, suggest active production of raffinose (galactose addition to sucrose via galactinol), a storage carbohydrate which accumulated. More uronic acids (2.5-fold) and amino acids (glutamine, tyrosine, alanine, γ-aminobutyric acid) were also found in the MYB4-OX methanol extractives (Table 1). Accumulation of most of the soluble sugars measured, coupled with the decline in monolignols, related upstream precursors, downstream lignans, and reduced lignin content with the overexpression of PvMYB4 suggests altered partitioning of carbon away from the lignin pathway (secondary metabolism), consequently benefitting primary metabolism.

### Changes in cell wall components in PvMYB4-OX switchgrass

PvMYB4-OX switchgrass transgenic lines have thinner stems with smaller vascular bundles [12], although there were no obvious differences in stem structure. The cell walls appeared to be thicker in the control lines based on staining of stem sections [12]. We measured the wall thickness of the parenchyma cells in the mature stem sections (E4I1 internode); the value for control plants was 4.21 ± 0.52 μm, compared with 1.85 ± 0.50 μm for the PvMYB4-OX transgenics (Student *t*-test E-value *p* = 6.0E-20).

**Figure 2:**
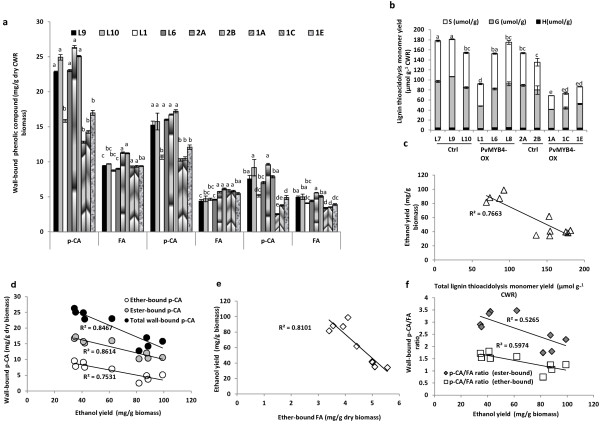
**Correlations between lignin content, wall-bound phenolics and SSF ethanol yield.** (**a**) The content of ester-linked, ether-linked and total wall-bound *p*-coumaric and ferulic acids in the cell wall residues (CWR) of switchgrass whole tillers. (**b**) Lignin composition of control and PvMYB4-OX switchgrass whole tillers determined by thioacidolysis. Ctrl: control lines; S, syringyl unit; G, guaiacyl unit; H, *p*-hydroxyphenyl unit. (**c**-**f**) Correlations with SSF ethanol yield without pretreatment. (**c**) lignin content. (**d**) wall-bound *p*-coumaric acid. (**e**) ether-lined ferulic acid. (**f**) wall-bound p-CA/FA ratio. All data are means ± SE (n = 3). The letters indicates significant differences of total lignin content at the *p* < 0.05 level. Mean comparisons, based on mean separation test results, cannot be compared across variables in Figure 2a.

After removal of the methanol extractives, the wall-bound (ester- and ether-linked) phenolics in the cell wall residues were released by successive hydrolysis in 2 M NaOH at 37°C for 5 h, and 4 M NaOH with autoclaving for 2 h, respectively, and were then measured by HPLC. Levels of total wall-bound *p*-CA and FA, and ester-bound and ether-bound *p*-CA, were reduced in all PvMYB4-OX lines except for L6. There was also a slight reduction in ether-bound FA content in PvMYB4-OX lines in the ST2 background (Figure 2a). No changes were observed for ester-bound FA. Thus, both ester-bound and ether-bound *p*-CA/FA ratios were significantly reduced in lines highly over-expressing MYB4 (Additional file 1: Figure S2b).

Total lignin thioacidolysis yields were reduced by about 50% in L1 and about 20% in L6 ST1 lines (Figure 2b). The SSF ethanol yield, without pretreatment, showed a strong negative correlation with total lignin content (R^2^ =0.77) (Figure 2c), total wall-bound *p*-CA (R^2^ =0.85), ester-bound *p*-CA (R^2^ =0.86), ether-bound *p*-CA (R^2^ =0.75) (Figure 2d) and ether-bound FA (R^2^ =0.81) (Figure 2e), and a weak negative correlation with ester-bound *p*-CA/FA ratio (R^2^ =0.52) as well as ether-bound *p*-CA/FA ratio (R^2^ =0.60) (Figure 2f).

**Figure 3:**
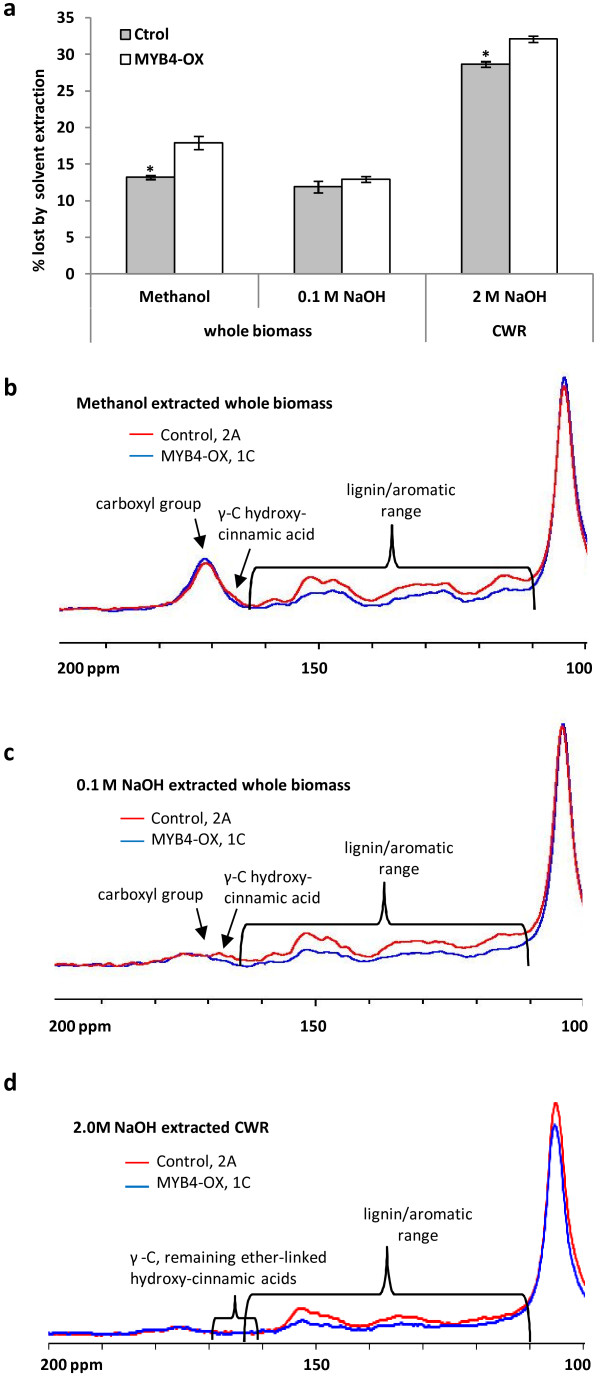
**Cell wall extractabilities and characteristics determined by solid-state**^**13**^**C CP/MAS NMR spectroscopy.** (**a**) Cell wall integrity of whole biomass or cell wall residues (CWR) determined by the percentage of mass lost under different extraction conditions. 0.1M NaOH, 0.1 M NaOH extraction overnight at 5°C after 91-92% methanol extraction overnight at 5°C. 2M NaOH: extraction of ester-linked wall-bound phenolics from CWR. *Asterisks indicate values that were determined by the Student *t*-test to be significantly different from their equivalent control (*p* < 0.05). All data are means ± SE (n = 3). (**b-d**) Solid-state 13C CP/MAS NMR spectra of CWRs of control (2A) and PvMYB4-OX (1C) biomass that had been extracted with methanol (**b**), 0.1M NaOH (**c**) or 2M NaOH (**d**). Whole biomass, non-extracted biomass from the whole tillers. CWR, cell wall residues of the whole biomass extracted by methanol: chloroform, methanol, methanol: H_2_O and H_2_O.

Biomass was extracted with methanol followed by 0.1 M NaOH at 4°C, or with methanol and chloroform followed by 2.0 M NaOH at 37°C to release the ester-linked wall-bound phenolics. Both methanol and alkaline hydrolysis removed more mass from PvMYB4-OX lines than from controls (Figure 3a). Extractive-free cell-wall residues were then characterized by solid-state ^13^C CP/MAS NMR (nuclear magnetic resonance) spectroscopy. Two control (2A and 2B) and five transgenic lines (1A, 1B, 1C, 1Dand 1E) from ST2 were analyzed and gave similar results; however, data are shown for only for 2A and 1C for figure clarity (Figure 3b-d). The lignin and aromatic region was assigned to the 110–165 ppm region, which reflects free monomers or wall attached lignin and hydroxycinnamate conjugates; this region was remarkably reduced in the PvMYB4-OX lines (Figure 3b-d), in agreement with the chemical analysis. Compared to the methanol extraction, which removes non-wall-attached phenolics and other free compounds (Figure 3b), the following dilute base extraction (Figure 3c) removed most (if not all) of the sugar acetylation (largely from carboxyl groups of hemicelluloses) as shown by the almost complete disappearance of the hemicellulose acetyl carbonyl signal (168–177 ppm). This then revealed a clear difference in the transgenic versus control line in the region of 162–170 ppm (peak 165 ppm) (Figure 3c). The 162–170 ppm region can be seen as a slight shoulder in the control after methanol extraction (Figure 3b), and corresponds to C_γ_ = O side chain or the C4 of the phenyl ring of wall-bound hydroxycinnamates. The reduced signal in this region in PvMYB4-OX lines indicates a decrease of ester-bound hydroxycinnamates.

An obvious signal reduction in the aromatic regions of solid-state NMR spectra of methanol extracted and dilute base extracted residues was observed for MYB4-OX lines (Additional file 1: Figure S3). The region 146–153 ppm is assigned to the C3 of both mono and di-methoxylated aromatic rings, and also to the C4 from mono-methoxylated aromatics and C5 from di-methoxylated aromatics. The region at 125–135 ppm can be largely attributed to aromatic carbons which have a carbon attached, and the alpha and beta carbons on the propenyl side chains of the aromatic ring. Decreased signals in these two regions are possibly due to de-esterification of lignin or cinnamaldehydes in the PvMYB4-OX lines.

There were small changes in total sugar content of PvMYB4-OX whole biomass (Additional file 1: Table S1). The major monosaccharides released by acid hydrolysis were glucose, xylose and arabinose, which represent, respectively, about 60%, 32% and 4-5% of the total sugars of the whole biomass. There were no significant differences in total sugar content of cell-wall residues from PvMYB4-OX and control lines after removal of soluble sugars and starch from whole biomass (Additional file 1: Table S2). About 7–9 mg of total pectin was extracted per gram of alcohol insoluble cell wall residue. Only 25% of this was extractable by water and sodium acetate/EDTA solution, and about 85% (wall-bound pectin) was released by 0.1M HCl at 100°C for 1 h (Additional file 1: Table S3. More pectin was released from MYB4-OX lines than from controls (Additional file 1: Table S3). Thus, down-regulation of lignin content in PvMYB4-OX lines leads to increased soluble and wall-bound pectins in the cell walls (Additional file 1: Table S3).

### Overexpression of PvMYB4 reduces lignin size and internal linkages

Lignin molecular weight reduction is linked to reduced recalcitrance of low lignin alfalfa (*Medicago sativa*) [14]. Isolated lignins were prepared from two control and two MYB4-OX lines, and their molecular weights measured by gel permeation chromatography (GPC) (Additional file 1: Figure S4). The average molecular weights of the isolated lignins were lower in the PvMYB4-OX lines (1C and 1D), 4,400-4,900 Da as compared to 5,300-5,500 Da in control lines (2A and 2B). These changes are much smaller than reported in low lignin alfalfa [14].

**Figure 4:**
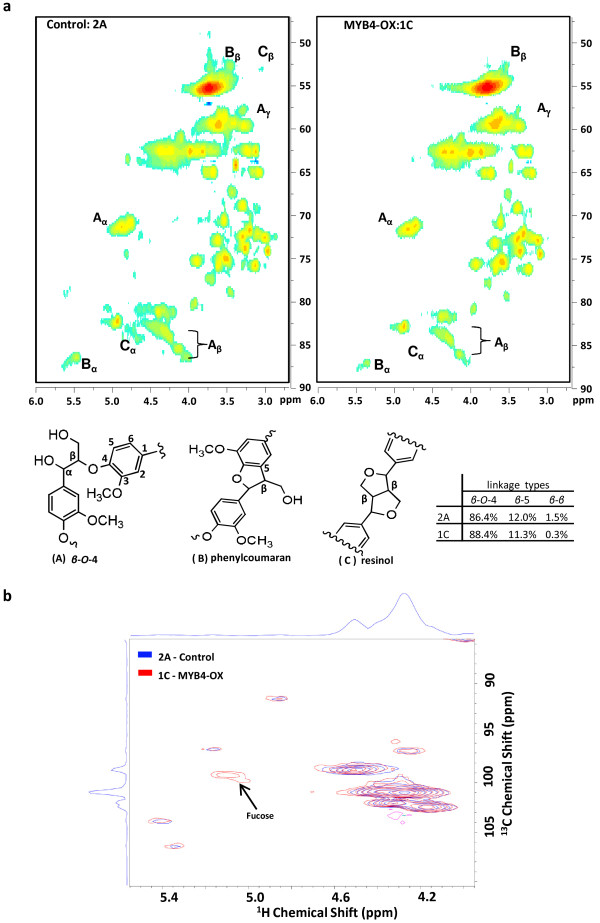
**Partial HSQC spectra showing the side-chain C-H correlations from the three main linkages** (***β***-***O***-**4**-, ***β*-5-, and *β***-***β***) **in lignins and presence of wall-associated fucose.** (**a**) Partial HSQC spectra of isolated lignins. The carbon positions in aromatic ring and side-chain are labeled on the molecular structures of the A, B and C types of linkages. The insert table shows the relative content of different linkage types. (**b**) Partial 2D HSQC NMR analysis of gel-state CWR showing increased wall-associated fucose in PvMYB-OX. Red, control 2A. Blue, PvMYB4-OX.

To check lignin inter-unit linkages, isolated lignins were analyzed by 2D heteronuclear single quantum coherence (HSQC) NMR based on two-dimensional chemical shifts of protons and carbon linkages. Spectra were collected on controls 2A and 2B and transgenic lines 1C and 1D but data are only presented for 2A and 1C for simplicity and consistency with other figures. The aromatic regions of the ^13^C-^1^H HSQC spectra showed no significant differences in aromatic C-H correlations between PvMYB4-OX and control, indicating that the basic monolignol constituents of the lignins are the same (Additional file 1: Figure S5). However, in the aliphatic regions of the spectra, the C-H correlations of the lignin side chains in *β*-*β* linkages (resinols) were decreased in PvMYB4-OX lines, whereas the other two major linkages, *β*-*O*-4 and *β*-5 (phenylcoumaran) were relatively unchanged (Figure 4a). Gel-state 2D HSQC NMR spectroscopy also revealed that PvMYB4-OX lines have about a five-fold higher level of fucose residues in the cell walls (Figure 4b).

### Reduced association of xylan and pectins with lignin in PvMYB4-OX switchgrass

**Figure 5:**
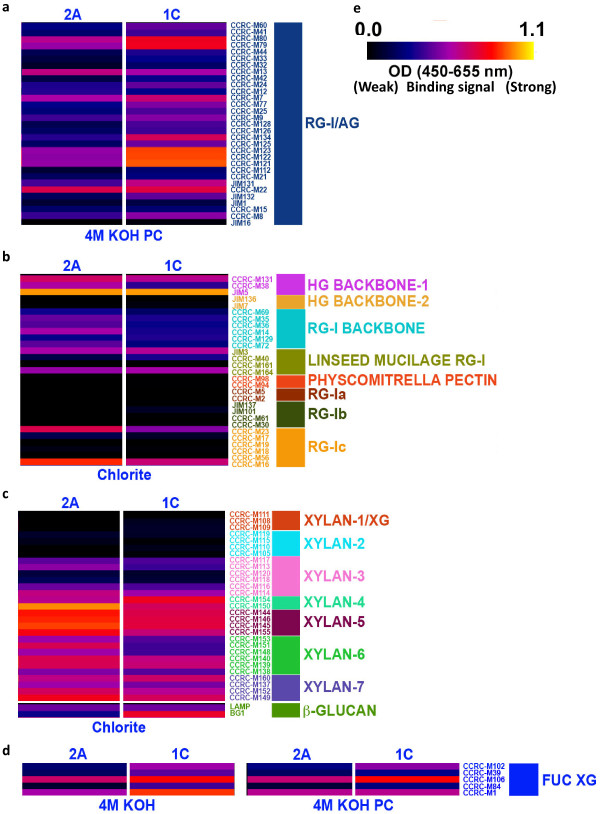
**Glycome profiling heatmaps of specific cell wall extracts showing areas that are highlighted in Additional file **1,**with indication of antibodies.** The white, blue, yellow and green colored boxes highlighted in Additional file 1 are enlarged in (**a-d**) to show differences in antibody binding signals. (**e**) Heatmap scale. The black, blue, red and yellow colors indicate the binding intensities of groups of plant glycan-directed monoclonal antibodies (with black color depicting no binding and bright yellow depicting strongest binding) that are selective for the different cell wall polysaccharides as labeled on the right-hand side of the figure.

We conducted glycome profiling analyses [15] of sequential extractions of PvMYB4-OX and control cell wall residues to assess the strengths of association of various polysaccharide polymers within the cell walls (Additional file 1: Figure S6). Oxalate and carbonate remove mainly pectins and a small portion of hemicellulose from the walls, and the following 1M and 4M KOH treatments extract most of the tightly bound hemicelluloses and pectin. Chlorite removes a significant portion of the lignin, and the post chlorite 4 M KOH extraction releases additional hemicellulose and pectin components. The extracts were then screened by enzyme-linked immunosorbent assay using a comprehensive set of plant glycan-directed monoclonal antibodies (mAbs) (Additional file 1: Table S4) that recognize diverse epitopes on most of the major plant cell wall polysaccharides [15,16]. The glycome profiles (represented as heatmaps) were largely similar for control and PvMYB4-OX lines, the most notable differences being in the chlorite and post-chlorite 4M KOH extracts (Additional file 1: Figure S6). Increased binding intensities in the 4M KOH PC extracts of MYB4-OX lines were for mAbs that recognize pectic arabinogalactan (RG-I/AG) epitopes (Additional file 1: Figure S6, white boxes and Figure 5a). This suggests that lignin in the wild-type secondary cell wall blocks accessibility to such pectic polysaccharides, which are traditionally considered as major components of the primary cell wall and middle lamella. Alternatively, more RG-I/AG polysaccharides are present in the walls of PvMYB4-OX lines. Reduced signals for the pectic RG-Ic, RG-I and HG backbone groups of antibodies were observed in the chlorite extracts from MYB4-OX lines (Additional file 1: Figure S6, blue boxes and Figure 5b). These data reveal associations of pectic polysaccharides with lignin in switchgrass, and the reduced lignin level in PvMYB4-OX lines potentially reduces such associations.

Less xylan epitopes were released during the chlorite extraction in the MYB4-OX lines (Additional file 1: Figure S6, yellow boxes and Figure 5c), suggesting less xylan cross-linking/association with lignin. The chlorite treatment is unlikely to affect other wall components, and hence the release of the carbohydrates by this treatment will arise due to the destruction of the lignin component that ties these epitopes into the wall. Note that only a subfraction of these epitopes is released in the chlorite step; other subfractions of these polymers are not tied to lignin and are released in other extracts.

The chlorite extract of the PvMYB4-OX lines also showed increased binding to the mAb BG-1, which is specific for hemicellulosic β-1,3-1,4 glucan [17] (Additional file 1: Figure S6, yellow boxes and Figure 5c). An increase of fucosylated xyloglucan signal was also revealed by the binding of antibodies such as CCRC-M1, CCRC-M102, and CCRC-M106 in both the 4 M KOH and 4 M KOH PC extractives of the MYB4-OX lines (Additional file 1: Figure S6, green boxes; Figure 5d). The fucose in xyloglucan is (as far as is known to date) located in the terminal position on the side-chains [18] and the antibodies are specific for the fucose in that position [19], thus, it is likely that the antibodies are indeed detecting an increase in fucose level rather than an unmasking of the epitope. This is consistent with the increased wall-associated fucose observed by NMR analysis being due to increased fucosylated xyloglucans in PvMYB4-OX cell walls.

### An improved system for high bioethanol production

Overexpression of PvMYB4 reduces the lignin content of switchgrass by 60-70% and increases sugar release efficiency approximately 3-fold without acid pretreatment [12]. This translates into a 2.6-fold increase in ethanol yield using yeast-based SSF without pretreatment. PvMYB4-OX switchgrass produces approximately 1.8-fold more ethanol than COMT-RNAi switchgrass [9] under the same fermentation conditions. The COMT-RNAi transgenic lines require only 25-30% the level of cellulase for equivalent ethanol fermentation compared to control switchgrass, with an estimated cost reduction for biomass processing of 21-25% for enzyme alone after excluding biomass and capital charges [9]. Based on the same calculations, PvMYB4-OX lines could save up to 45% of enzyme costs alone. Without a consolidated bioprocessing fermentation method, the minimum ethanol selling price (MESP) from switchgrass feedstock is $1.42-2.91 /gallon [20]. The estimated enzyme cost savings from use of PvMYB4-OX transgenic switchgrass will give $0.78-1.60 /gallon MESP which essentially meets the US Department of Energy’s $1.07/gallon target for 2012.

Fermentation inhibition by low molecular-weight compounds is a critical concern when processing lignin down-regulated biomass [21]. Increased levels of phenolic aldehydes and acids contribute to inhibition of microbial growth during fermentation of COMT-RNAi switchgrass [11]. In contrast, levels of monolignols, phenolic aldehydes, and phenolic acids are all reduced in PvMYB4-OX switchgrass lines, consistent with the improved yeast-based SSF fermentation results.

### New insights into recalcitrance of lignocellulosic feedstocks

Multiple factors may contribute to the recalcitrance of lignocellulosic feedstocks towards chemical treatments and/or enzymes, many of which are related to the presence of lignin in cell walls [22]. SSF ethanol yields negatively correlate with total lignin content, wall-bound *p*-CA (both ester-linked and ether-linked), ether-linked FA, and ester-linked *p*-CA/FA ratio in switchgrass. A decreased ester-linked *p*-CA/FA ratio has been associated with increased forage digestibility in barley [23] and increased sugar release efficiency in switchgrass [24]. FA serves as a bridge between lignin and hemicellulose [25], and ferulate-arabinoxylan esters can form ether linkages with lignin polymers [26]. The reduced level of ether-bound FA in MYB4-OX switchgrass suggests a looser wall association between lignin and arabinoxylans, as confirmed by extractability studies and glycome profiling. Reduced lignification or ferulate-lignin cross-linking are also important for improved fiber fermentability in maize suspension cells [27]. Overall, our data suggest that reduced lignin content, polymer size and changes in inter-unit linkages all likely contribute to the reduced recalcitrance of PvMYB4-OX lines.

Fewer pectic epitopes (RG-Ic, RG-I backbone and HG backbone-1 groups) are released from PvMYB4-OX wall residues during chlorite extraction. This suggests that specific sub-populations of these pectic polysaccharides may directly link/associate with lignin. Older literature suggests that pectic arabinogalactans can be removed concurrently with lignin during the delignification of lupin by chemical treatments [28,29]. A study in alfalfa suggested that the deposition and distribution of pectin corresponded to the deposition patterns of lignin in the middle lamella [30], where much of the pectin in the cell wall is located and lignification is initiated [31]. A recent study also suggests the presence of critical associations between lignin and pectins in *Populus* biomass, where hydrothermal pretreatment disrupts lignin-polysaccharide interactions together with a loss of pectins and arabinogalactans [32]. Although a pectin-hemicelluose-cellulose network has been widely accepted, direct lignin-pectin linkages/interactions should be further investigated in view of their potential contribution to recalcitrance.

Lignin and wall-bound phenolics are not the only factors impacting recalcitrance in switchgrass. Glycome profiling and NMR revealed increased levels of wall-associated fucose, possibly in fucosylated xyloglucans, in PvMYB4-OX lines. Fucosylated cell wall components in plants include glycolipids, O- and N-glycoproteins and polysaccharides such as xyloglucans and rhamnogalacturonans (RG). The glycolipids will be removed by methanol extraction and thus do not contribute to the fucose measured in the present study. Cell wall glycoproteins can form ether and aryl linkages through tyrosine, lysine and sulfur-containing amino acids with hydroxycinnamic acids esterified to polysaccharides in the cell wall. The fucosyl residues in RG-II and xyloglucan are important for the strength of load-bearing elements in cell walls [33,34]. Fucosylated xyloglucans are thought to have interconnections with the cellulosic matrix [35], and *in vitro* binding assays and computer modeling suggest that the fucosyl groups of xyloglucan may stabilize a xyloglucan conformation and help the polysaccharide to bind more tightly to cellulose in the wall matrix [36,37]. Fucosylated oligosaccharides derived from xyloglucans may also act as signal molecules in plant-pathogen interactions or plant growth regulation [38,39]. The increased fucose content of RG-II and xyloglucan in PvMYB4-OX lines might compensate for the mechanical weakness caused by the reduced lignin levels in the cell walls, explaining why PvMYB4-OX tillers do not show severe lodging when grown in the greenhouse.

## Conclusions

The concept of increased saccharification efficiency and ethanol yield through down-regulation of single lignin biosynthetic genes has been proven successful, while also creating problems, including the accumulation of upstream phenolic metabolites that are fermentation inhibitors. Our results demonstrate that an alternative approach, the overexpression of a general transcriptional repressor of the phenylpropanoid/lignin biosynthesis pathway, can reduce carbon flux into the lignin biosynthetic pathway and produce a bioenergy crop with reduced cell wall recalcitrance, slightly increased polysaccharide content and reduced levels of phenolic fermentation inhibitors. The very large improvement in ethanol yield, proportional to the dramatic reduction of recalcitrance, makes MYB4-OX switchgrass an excellent model system for understanding the chemical basis of recalcitrance, and for the development of economically viable lignocellulosic feedstocks for biofuel production. It is important to note that selection of specific transgenic events for incorporation in breeding programs is based on multiple considerations. Important in the lignin modification field is the trade-off between reduced recalcitrance and biomass yield. In this respect, line L6 (intermediate high overexpression of PvMYB4) grows much better than more highly overexpressing lines. Although we see a strong correlation between wall-bound phenolic levels and recalcitrance (determined as final ethanol yield) based on our whole population of transgenics, there is no change in wall-bound phenolic levels in line L6, although this line does show improved ethanol yields.

## Materials and methods

### Plant materials

*Agrobacterium*-*mediated* switchgrass transformation used constructs [12] and methods [40] described previously. The ST1 and ST2 lines were provided by Dr Zeng-Yu Wang, Noble Foundation. L7, L9 and L10 are transgenic control lines in the ST1 background. L1, L2, L4, L6 and L8 are MYB4-OX lines in the ST1 background. Lines 2A and 2B are vector controls for 1A-E (MYB4-OX) lines in the ST2 gene background.

All plants were grown under greenhouse conditions as described [24]. Harvested tillers (at R1 stage) were either frozen and milled by a freezer mill (SPEX SamplePrep, Metuchen, NJ) in liquid nitrogen for genomic DNA or RNA isolation, or dried at 40°C for one week then milled in a Thomas Wiley® Mini-Mill (Thomas Scientific, Swedesboro) through a 0.8 mm screen to 20 mesh for chemical analysis and ethanol fermentation tests. Samples for analysis of lignin content, wall-bound phenolics and solid-state NMR were further milled to 60 mesh size.

### Measurement of lignin,phenolic and pectin content

Lignin content and composition of cell wall residues was determined by thioacidolysis followed by GC-MS as described previously [24]. Soluble phenolics were extracted from 30 mg of freeze-dried tissue powder with 50% (v/v) methanol and assayed by HPLC, which reveals chlorogenic acid derivatives as the majorsoluble phenolics. Total soluble phenolic levels were assayed with Folin-Ciocalteu reagent, and wall-bound phenolics were analyzed as described previously [24].

For determination of pectin, plant material was ground in liquid N_2,_ homogenized with 2 volumes of 80% ethanol, and incubated overnight at 4°C. The homogenate was centrifuged at 3,000 rpm for 5 min and the alcohol insoluble cell wall residue (AIR) washed twice with 20 ml of absolute ethanol and dried under N_2_. One hundred mg of AIR were extracted sequentially with water (20 ml, shaken overnight at room temperature), 0.05 M sodium acetate containing 0.04 M EDTA, pH 4.5 (20 ml, shaken for 4 h at room temperature) and 0.05 M HCl (20ml, incubated at 100°C for 1 h). Two hundred μl of supernatant from the different fractions was further hydrolyzed with 900 μl of H_2_SO_4_/sodium tetraborate reagent at 100°C for 5 min. The reaction was stopped on ice and the pectin content was determined by the m-hydroxydiphenyl method [41] with galacturonic acid as standard.

### Quantitative saccharification, pretreatment and ethanol fermentation

Quantitative saccharification assays were as described in ASTM E 1758–01 (ASTM 2003) and HPLC method NREL/TP 51–42623. Hot water pretreatment was conducted using the tubular batch method [42], except only one sand bath (Omega FSB1, Techne Co., Princeton, NJ) was used to heat the 4 × 0.5 inch pretreatment tubes.

Simultaneous saccharification and fermentation (SSF) with *Saccharomyces cerevisiae* D5A (ATCC 200062) was performed as described in Fu et al. [9] with the exception that Accellerase 1500 enzyme (final concentration of 11.5 FPU per gram of cellulose), kindly provided by Genencor International, Inc., was used instead of Spezyme CP and Accellerase BG.

### Solvent extraction of switchgrass biomass for solid-state NMR

Sequential extraction was performed as reported previously [14]. Ester-linked wall-bound phenolics were extracted as described previously [24]. The pellet residue was washed with water until the supernatant was neutral. The solids were then freeze-dried and weighed for solid-state NMR analysis.

### Gel permeation chromatography (GPC) of lignin

Ball-milled lignin was isolated from extractives-free switchgrass as described previously [43]. The yields were 1.022% (1C), 1.361% (1D), 2.223% (2A) and 2.286% (2B). *GPC*: Isolated lignin samples were acetylated and GPC analysis performed using an Agilent HPLC with three polystyrene-divinyl benzene GPC columns (Polymer Laboratories, 300 × 7.5 mm, 10 μm beads) having nominal pore diameters of 10^4^, 10^3^, and 10^2^ Å. The eluent was THF, the flow rate 1.0 ml/min, the sample concentration was ~2 mg/ml and an injection volume of 25 μl was used. The HPLC was attached to a diode array detector measuring absorbance at 260 nm (band width 40 nm). Polystyrene calibration standards were used with molecular weights ranging from 580 Da to 2.95 million Da. Toluene was used as the monomer calibration standard.

### Solid, gel and solution-state NMR

Cross-polarization/magic angle spinning (CPMAS) spectra were collected as described previously [14] with slight modifications: A 7 mm ZiO_2_ rotor was loaded with approximately 75 mg of dried biomass ground to 60 mesh. CPMAS NMR spectra were collected on a Bruker DSX 200 spectrometer equipped with a 7 mm CPMAS probe and a 4.7 T magnet (^1^H = 200.1 MHz and ^13^C = 50.32 MHz). A ramped CP pulse with ^1^H and ^13^C fields matched at 48 kHz was applied with a contact pulse of 2 ms. An acquisition time of 0.051 s and a recycle delay of 1s were used with 2 k points collected and averaged over 40k scans for each spectrum with MAS = 7 kHz.

Samples of whole biomass and isolated lignin were prepared for 2D gel state NMR by suspending 20–30 mg of material in 0.5 ml of DMSO-d_6_ in a 5 mm NMR tube. Samples were then sonicated for 2h (whole biomass) or 30 min- 1 h (isolated lignin).

Gel-state ^1^H-^13^C HSQC spectra were collected on a Bruker Avance III 600MHz spectrometer with a 5 mm TCI cyroprobe. HSQC spectra were acquired with a sweep width of 15 ppm, 1024 data points, and an acquisition time of 57 ms in the F2 dimension. For the F1 dimension a sweep width of 166 ppm was used with 256 increments. The recycle delay was set to 1.5 s and 128 scans were collected for each increment for a total experiment time of 14.5 h.

For 2D HSQC NMR spectral analysis, lignin samples were isolated according to modified literature methods [44-46]. In brief, 20 mesh switchgrass biomass was Soxhlet-extracted with benzene-ethanol (2:1, v/v) for 24 h to remove extractives. The extracted wall residue was then milled in a porcelain jar (1 l) with ceramic balls using a rotatory ball mill running at 96 rpm under nitrogen for 120 h. The ball milled powder was then suspended in 20 mM sodium acetate, pH 5.0. A mixture of Cellulysin cellulase (EC 3.2.1.4, Calbiochem, http://www.calbiochem.com), Cellobiase (Novozyme 188 from *A*. *niger*) and xylanase was added and the slurry incubated with shaking at 200 rpm and 37°C for 48 h. The digested cell wall fractions were then extracted twice with dioxane-water (96:4, v/v) under stirring for 24 h. The extract was centrifuged and the supernatant evaporated under reduced pressure, and freeze-dried. The resulting crude lignin-enriched samples were washed with deionized water and purified by liquid-liquid extraction [44] for NMR characterization.

### Glycome profiling

Glycome profiling was carried out by enzyme-linked immunosorbent assays of cell wall extracts using a large collection of plant glycan-directed monoclonal antibodies (http://www.wallmabdb.net) as described previously [15,16] (Additional file 1: Table S4).

### Metabolite profiling

Metabolite profiling of methanol extracts was performed as reported previously [11] with modifications: Ten ml of the extracts were dried under nitrogen. Sorbitol (15 μg) was added as internal standard, and the extracts were silylated for 2 days as described previously [11], and 0.5 μl of the 1-ml reaction volume was analyzed by GC-MS.

### Statistical analyses

Metabolite data were averaged by control and PvMYB4-OX lines. Five biological replicates were analyzed for the PvMYB4-OX line and two for the control line, and two technical replicates were averaged for each sample. *p*-Values were determined by Student’s *t*-test (Microsoft Office Excel 2007) and *p* < 0.05 (indicated by asterisks in figures) considered as indicating significant differences. Multiple comparisons were done with SAS software (SAS Institute Inc., Cary, NC). Tukey’s honestly significant difference test was used when the null hypothesis was rejected (*p* < 0.05). Means with the same letter, within each variable, are not significantly different at *p* < 0.05.

## Abbreviations

AG: Arabinogalactan; AIR: Alcohol insoluble residue; CA: *p*-coumaric acid; CAD: Cinnamyl alcohol dehydrogenase; CP/MAS NMR: Cross polarization/magic angle spinning nuclear magnetic resonance; COMT: Caffeic acid 3-*O*-methyltransferase; DMSO: Dimethylsulfoxide; EDTA: Ethylenediaminetetraacetic acid; FA: Ferulic acid; GC-MS: Gas chromatography–mass spectrometry; GPC: Gel-permeation chromatography; HG: Homogalacturonan; HMF: 5-hydroxymethylfurfural; HPLC: High performance liquid chromatography; HSQC: Heteronuclear single quantum coherence; mAbs: Monoclonal antibodies; MESP: Minimum ethanol selling price; MYB: Myeloblastosis family; PvMYB4-OX: Switchgrass plants overexpressing the switchgrass *MYB4* gene; RG: Rhamnogalacturonan; RNAi: Ribonucleic acid interference; SSF: Simultaneous saccharification and fermentation; THF: Tetrahydrofuran.

## Competing interest

All authors declare that they have no competing interests.

## Authors’ contributions

HS and CRP generated the plant materials. HS performed molecular biology analyses, measured wall-bound phenolic compounds, assisted in coordination of the study, and helped draft the manuscript. CRP, JRM and CNC planned and performed the fermentation studies. AZ, EG, RK, YP, RS, AR, FC and MD performed analytical studies on plant cell walls, including use of NMR to characterize extractives and wall-associated components. NLE and TJP performed and interpreted metabolomics analysis. SP and MGH performed and interpreted glycome profiling. RAD conceived of the study, participated in its design and coordination, and drafted the manuscript. All authors read and approved the final manuscript.

## Supplementary Material

Additional file 1 Supporting figures and tables. Click here for file
