# Supplementary material for: Enhanced characteristics of genetically modified switchgrass (Panicum virgatum L.) for high biofuel production
Source: Biotechnol Biofuels. 2013 May 7;6:71. doi: 10.1186/1754-6834-6-71 (PMC3652750; doi:10.1186/1754-6834-6-71)
Supplement: Additional file 1 — Supporting figures and tables. [file 1754-6834-6-71-S1.pdf]

SUPPORTING FIGURES AND TABLES :

Enhanced characteristics of genetically modified switchgrass (*Panicum virgatum* L.) for high biofuel production†

Hui Shen,[af]* Charleson R. Poovaiah,[bf]* Angela Ziebell,[cf] Timothy J. Tschaplinski,[f] Sivakumar Pattathil,[df] Erica Gjersing,[cf] Nancy L. Engle,[f] Rui Katahira, [cf] Yunqiao Pu,[ef] Robert Sykes,[cf] Fang Chen,[af] Arthur J. Ragauskas,[ef] Jonathan R. Mielenz,[f] Michael G. Hahn,[df] Mark Davis,[cf] C. Neal Stewart, Jr.,[bf] Richard A. Dixon,[‡af]

[a]*Plant Biology Division, Samuel Roberts Noble Foundation, 2510 Sam Noble Parkway, Ardmore, Ok, 73401, USA. E-mail: radixon@noble.org; Fax:+580-224-6602; Tel:+590-224-6601*
[b]*Department of Plant Sciences, University of Tennessee, 2431 Joe Johnson Dr., Knoxville, TN, 37996, USA.*
[c]*National Renewable Energy Laboratory, 1617 Cole Blvd., Golden, CO,80401, USA.*
[d]*Complex Carbohydrate Research Center, University of Georgia, 315 Riverbend Rd., Athens, GA, 30602, USA.*
[e]*School of Chemistry & Biochemistry, Georgia Institute of Technology, 901 Atlantic Drive, Atlanta, GA, 30332, USA.*
[f]*BioEnergy Science Center (BESC), Oak Ridge National Laboratory, Oak Ridge, TN,37831, USA.*

(* these authors contributed equally to this work)

Fig.S1. Genomic DNA PCR and qRT-PCR for PvMYB4-OX lines in the Alamo ST1 background.

(a) The *pANIC2B-PvMYB4* construct used for switchgrass transformation. *OsAct1*, promoter of rice *Actin1* gene; *rubi3*, promoter of *rubisco*3 gene; *ZmUbi1*, promoter of maize *ubiquitin 1* gene. *hph*, hygromycin resistance gene for transformation selection marker. LB and RB, left and right borders of the T-DNA sequences. The double arrows show the primer pairs used for genomic DNA PCR testing. HPH, hygromycin-specific gene primer pairs; Gene specific, primer pairs covering the ZmUBI1 promoter region and PvMYB4 gene specific region.

(b) Genomic DNA PCR. L7, L9, L10 and ST1 are transgenic and non-transgenic control lines.

(c,d) qRT-PCR analysis showing (c) *PvMYB4* transgene transcripts in control and PvMYB4-OX transgenic switchgrass and (d) endogenous *PvMYB4* transcripts; the endogenous gene is down-regulated by over-expression of the *PvMYB4* transgene in switchgrass. Primer pairs used were as been reported before[12]. Data are means ±SE (n=3). L1 (high overexpression), L6 (intermediate overexpression), L9 and L10 (transgenic controls) were chosen for further analysis.

(e) Visible phenotypes of control (Line 2A) and PvMYB4-OX switchgrass (Line 1C).

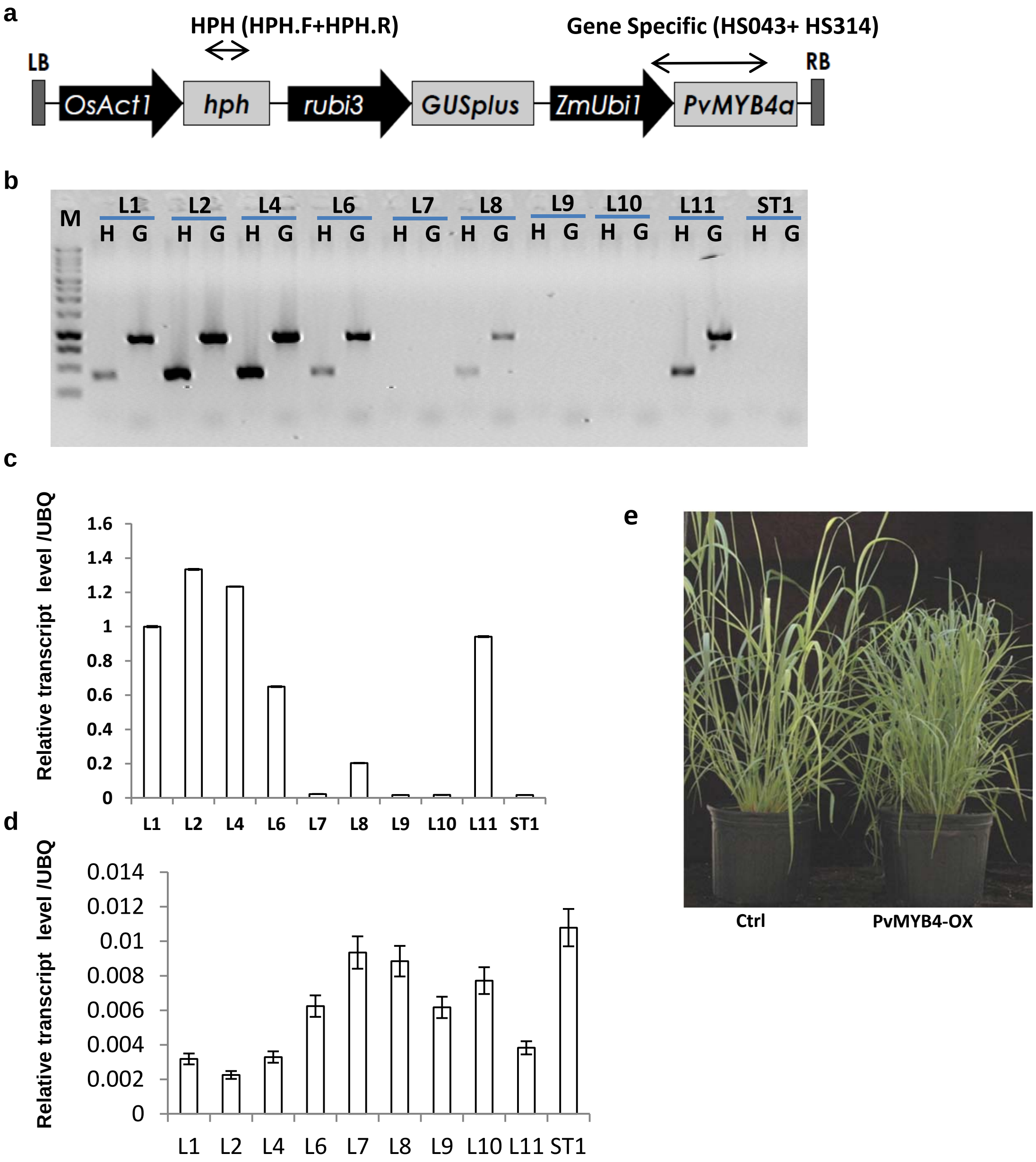

Fig. S2. PvMYB4-OX transgenic switchgrass lines have reduced levels of soluble phenolic compounds and reduced ester- and ether-linked *p*-coumaric/ferulic acid ratios.

(a) Levels of total soluble phenolic compounds as determined by reaction with Folin Ciocalteau reagent. (b) Levels of ester- and ether-linked wall-bound *p*-coumaric/ferulic acid (*p*-CA/FA) ratios. All data are means ±SE (n=3). Different letters on the bars of figures indicate significant differences at the $p<0.05$ level. Mean comparisons, based on mean separation test results, cannot be compared across variables in panel **b**. Lines L9, L10, L1 and L6 are in the Alamo ST1 genetic background, lines 2A, 2B, 1A, 1C and 1E in the Alamo ST2 genetic background.

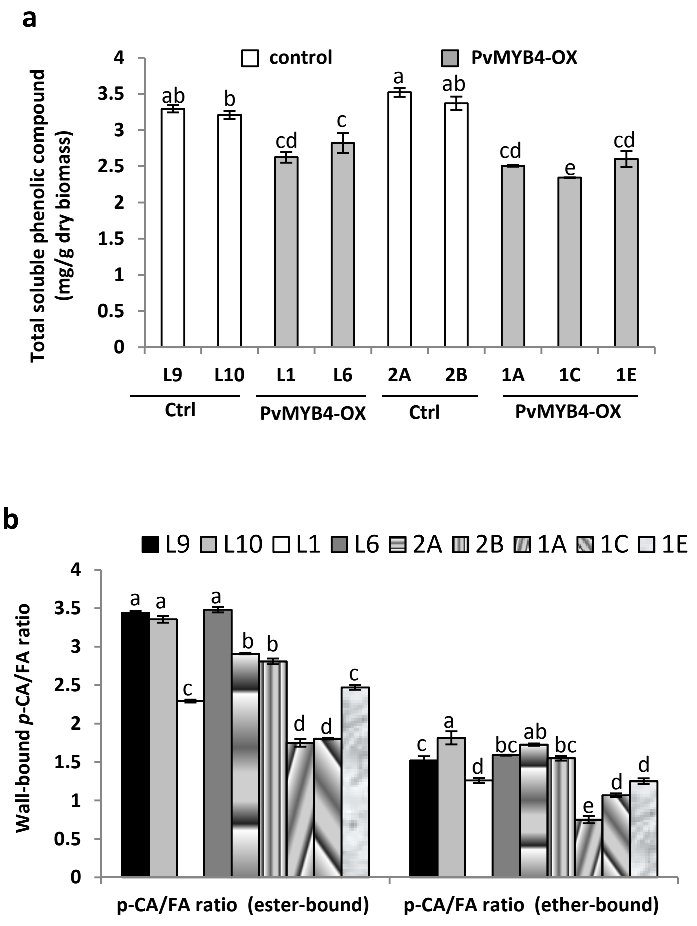

Fig. S3. Comparisons of methanol- (blue), 0.1 M NaOH- (red) and 2.0 M NaOH- (green) extracted cell wall residues determined by solid-state ^{13}C CP/MAS NMR spectroscopy.
Panels (a) and (b) show the NMR spectra for control line 2A and PvMYB4-OX line 1C, respectively.

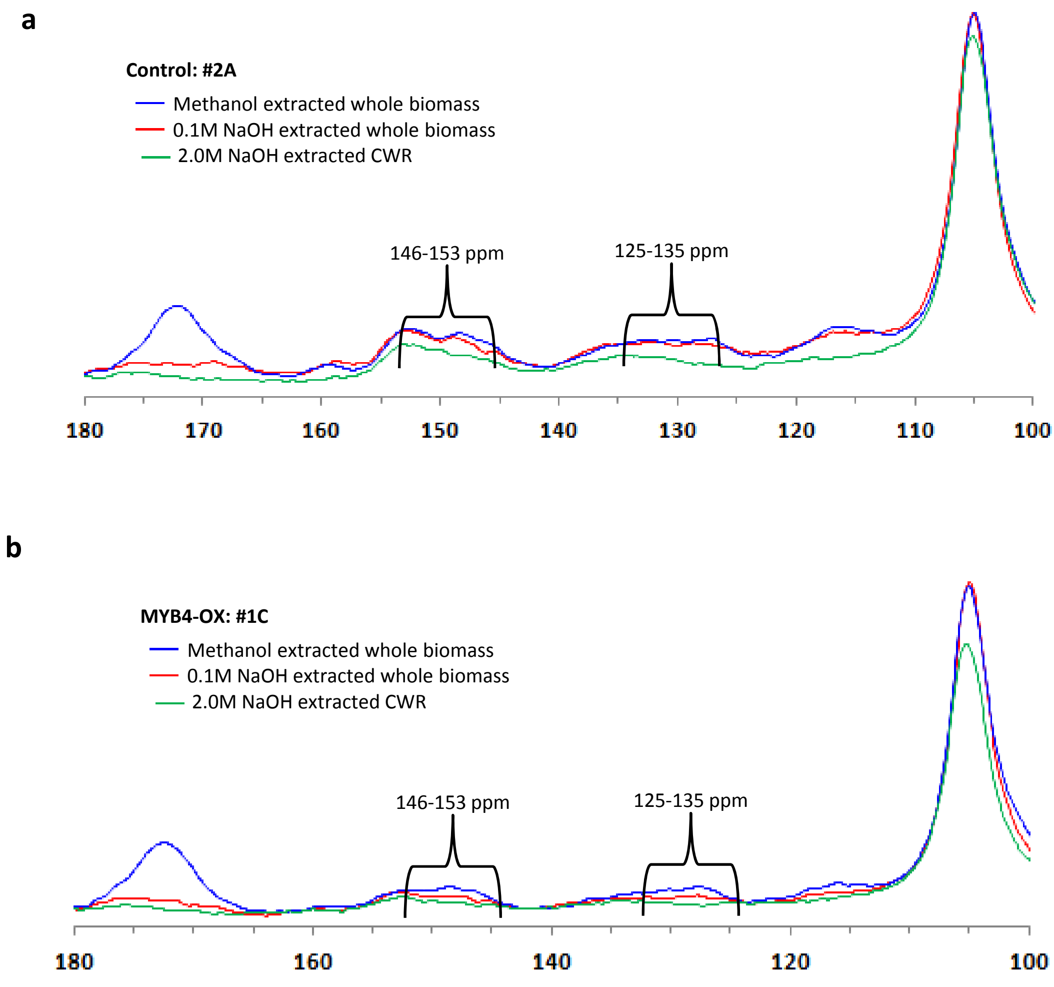

Fig. S4. Gel permeation chromatography analysis of isolated lignins from PvMYB-OX and control switchgrass.
Control lines: 2A and 2B. MYb4-OX lines: 1C and 1D.

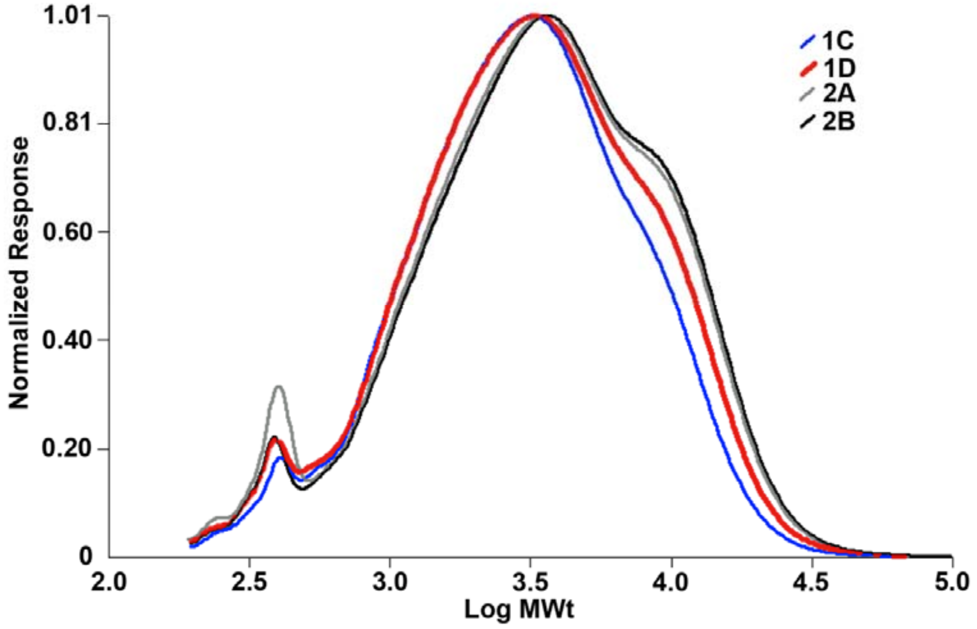

Fig. S5: Partial ^{13}C-^{1}H HSQC spectra of milled lignins showing the C–H correlations from aromatic rings of the structural units.
*p*CA, p-coumaryl unit. G, coniferyl unit. S, sinapyl unit.

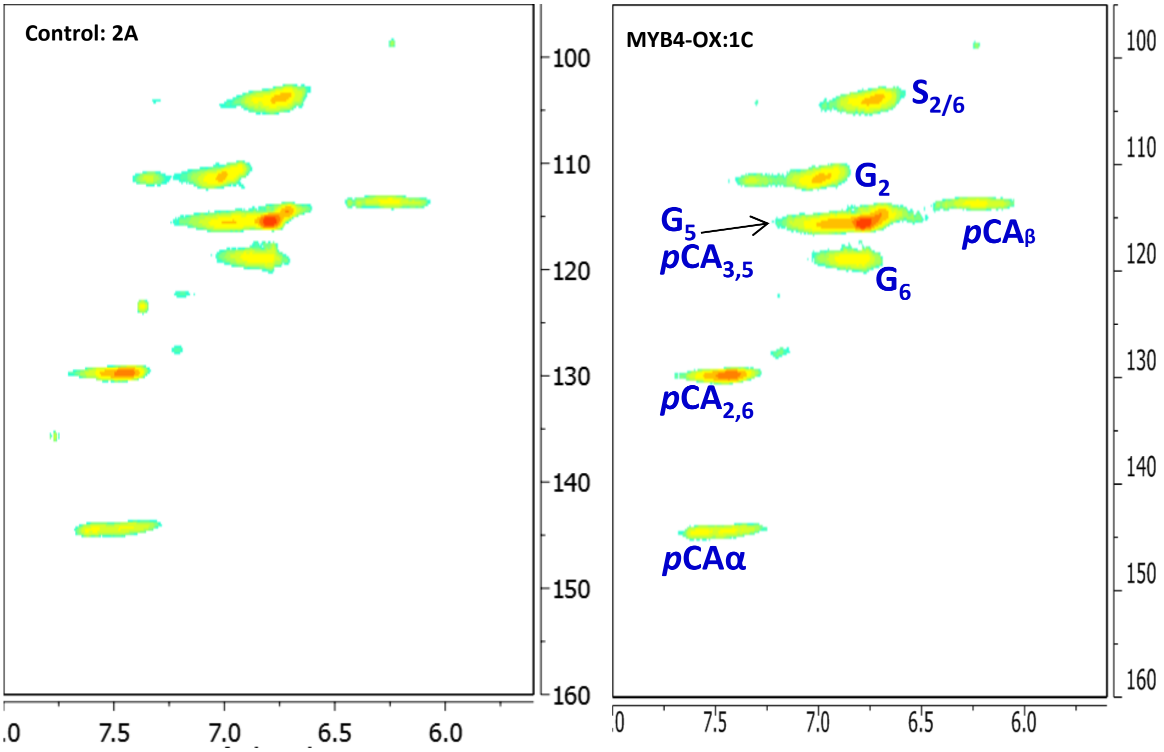

Aromatic regions of a ^{13}C-^{1}H HSQC spectrum switchgrass lignin.

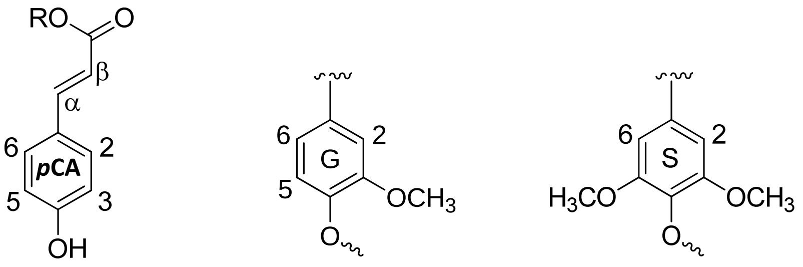

Fig. S6. Glycome profiling of extracts from alcohol insoluble cell wall residues from switchgrass.
Sequential extraction of alcohol insoluble cell wall materials from control and PvMYB4-OX transgenic lines was performed using a set of solvents with increasing strength, as indicated). The bars on the top panels of the figure indicates the amount of carbohydrates released by sequential extraction of the wall residues. The highlighted boxed areas show regions of difference between control and transgenics, and are featured in **Fig. 5** along with details of the antibodies.

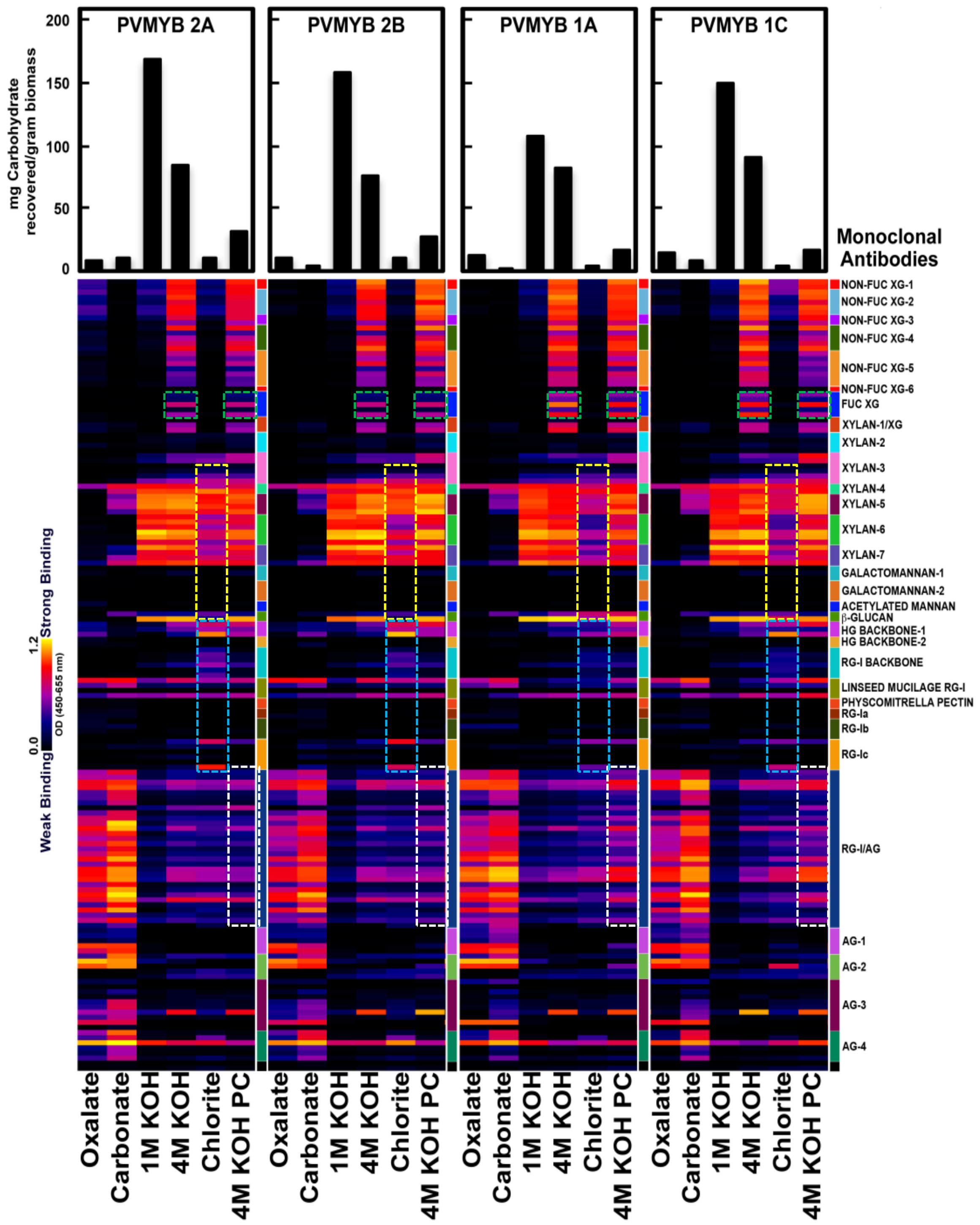

Table S1: Sugar composition (mg/g dry biomass) of whole tiller biomass determined by quantitative saccharification assay.

Lines	Glucose	Xylose	Galactose	Arabinose	Total sugars	Glucose (% of control)
Control (ST1)	339.2±7.1	158.0±10.1	20.8±0.5	29.4±0.4	549.3±17.1	100.0
L1	383.9±1.8*	161.8±1.1	28.2±0.5*	36.1±0.2*	613.1±3.2*	**113.2**
L6	319.7±12.2	129.0±13.5	16.9±0.7*	30.2±1.7	497.8±27.8	94.3
L8	351.3±1.4	157.8±4.2	16.6±0.1*	29.3±0.7	557.5±6.3	103.6
Control (ST2)	343.1±2.6	190.0±4.2	17.7±0.3	30.2±0.3	583.2±3.9	100.0
1A	320.2±25.4	142.6±24.7	25.0±1.6*	34.2±4.4	524.9±56.0	93.3
1B	359.0±1.4*	178.5±1.7*	24.7±0.1*	35.3±0.1*	601.1±3.0*	**104.6**
1C	353.6±3.3	169.1±0.9*	25.1±0.5*	34.5±0.2*	585.5±4.5	103.1
1D	344.1±3.3	179.5±2.4*	24.0±0.5*	34.1±0.2*	584.5±6.3	100.3
1E	371.8±3.7*	190.8±2.2	21.7±0.1*	34.0±0.4*	620.8±6.0*	**108.4**

Control for ST1 lines is the average of control lines L9 and L10. Control for ST2 lines is the average of control lines 2A and 2B. All data are means ±SE (n=6 for control lines and n=3 for transgenic lines). Asterisks indicate values that were determined by the Student *t*-test to be significantly different from their equivalent control (p<0.05).

Table S2: Levels of total sugars (mg/g dry cell wall residues) from control and PvMYB4-OX switchgrass after removal of soluble sugars and starch.

Lines	Glucose	Xylose	Galactose	Arabinose	Total sugars	Glucose(% of control)
Control (ST1)	468.2±8.6	254.0±2.2	12.8±0.6	24.1±0.7	759.0±9.6	100.0
L1	474.0±7.9	261.4±0.5	14.7±0.1	29.7±0.2	779.8±8.8	101.2
L6	477.1±2.1	261.2±1.3.	14.5±0.6	28.7±1.9	781.5±3.3	101.9
L8	472.3±5.1	259.2±0.2	13.0±0.7	25.8±2.3	770.3±2.3	100.9
Control (ST2)	426.1±5.7	270.5±3.2	12.1±0.3	25.0±0.9	733.7±8.0	100.0
1A	438.1±1.8	257.9±0.2	16.2±0.3	33.2±1.3	745.3±0.4	102.8
1B	443.9±1.8*	270.1±0.3	12.6±0.1	30.9±0.5	757.5±0.9	**104.2**
1C	448.2±0.0*	264.5±2.2	13.5±0.3	31.8±0.5	758.0±1.4	**105.2**
1D	444.8±7.2	274.5±4.3	13.1±1.1	31.3±3.9	763.7±6.5	104.4
1E	423.8±3.5	271.5±4.1	13.3±0.1	33.5±0.4	742.0±7.3	99.5

No significant differences among lines were detected. Control for ST1 lines is the average of control lines L9 and L10. Control for ST2 lines is the average of control lines 2A and 2B. All data are means ±SE (n=4 for control lines and n=2 for transgenic lines). Asterisks indicate values that were determined by the Student *t*-test to be significantly different from their equivalent control (p<0.05).

Table S3: Levels of pectin (mg/g alcohol insoluble cell wall residue) extracted from control and PvMYB4-OX switchgrass.

Lines	Water extracted	Na-AC/EDTA extracted	HCl extracted	Total pectin
2A (control)	0.73±0.05	0.33±0.14	5.93±0.08	6.98±0.18
1C	0.93±0.04 *	0.52±0.13 *	6.97±0.17 *	8.42±0.22 *
1D	0.77±0.07	0.50±0.13	7.10±0.27 *	8.37±0.34 *
L10 (control)	0.96±0.04	0.40±0.01	4.82±0.12	6.14±0.10
L1	1.04±0.06	0.49±0.23	7.13±0.08 *	8.66±0.32 *

All data are means ±SE (n=3). Asterisks indicate values that were determined by the Student *t*-test to be significantly different from their equivalent control (p<0.05).

Table S4:. Plant cell wall glycan-directed monoclonal antibodies (mAbs) used for glycome profiling analyses.

The groupings of antibodies are based on a hierarchical clustering of ELISA data generated from a screen of all mAbs against a panel of plant polysaccharide preparations[1,2] that groups the mAbs according to the predominant polysaccharides that they recognize. The majority of listings link to the Wall*Mab*DB plant cell wall monoclonal antibody database (http://www.wallmabdb.net) that provides descriptions of each mAb, including immunogen, antibody isotype, epitope structure (to the extent known), supplier information, and related literature citations.

1,. Pattathil S, Avci U, Baldwin D et al. A comprehensive toolkit of plant cell wall glycan-directed monoclonal antibodies. *Plant Physiol* 2010; **153**:514-525.

2, Pattathil S, Avci U, Hahn MG Immunological approaches to plant cell wall and biomass characterization: glycome profiling. *Methods Mol Biol* 2012, **908**: 61-72.

Glycan Group	mAb Names
Non-Fucosylated Xyloglucan-1	CCRC-M95, CCRC-M101
Non-Fucosylated Xyloglucan-2	CCRC-M104, CCRC-M89, CCRC-M93, CCRC-M87, CCRC-M88
Non-Fucosylated Xyloglucan-3	CCRC-M100, CCRC-M103
Non-Fucosylated Xyloglucan-4	CCRC-M58, CCRC-M86, CCRC-M55, CCRC-M52, CCRC-M99
Non-Fucosylated Xyloglucan-5	CCRC-M54, CCRC-M48, CCRC-M49, CCRC-M96, CCRC-M50, CCRC-M51, CCRC-M53
Non-Fucosylated Xyloglucan-6	CCRC-M57
Fucosylated Xyloglucan	CCRC-M102, CCRC-M39, CCRC-M106, CCRC-M84, CCRC-M1
Xylan-1/XG	CCRC-M111, CCRC-M108, CCRC-M109
Xylan-2	CCRC-M119, CCRC-M115, CCRC-M110, CCRC-M105
Xylan-3	CCRC-M117, CCRC-M113, CCRC-M120, CCRC-M118, CCRC-M116, CCRC-M114
Xylan-4	CCRC-M154, CCRC-M150
Xylan-5	CCRC-M144, CCRC-M146, CCRC-M145, CCRC-M155
Xylan-6	CCRC-M153, CCRC-M151, CCRC-M148, CCRC-M140, CCRC-M139, CCRC-M138
Xylan-7	CCRC-M160, CCRC-M137, CCRC-M152, CCRC-M149
Galactomannan-1	CCRC-M75, CCRC-M70, CCRC-M74
Galactomannan-2	CCRC-M166, CCRC-M168, CCRC-M174, CCRC-M175
Acetylated Mannan	CCRC-M169, CCRC-M170
β-Glucan	LAMP, BG1
HG Backbone-1	CCRC-M131, CCRC-M38, JIM5
HG Backbone-2	JIM136, JIM7
RG-I Backbone	CCRC-M69, CCRC-M35, CCRC-M36, CCRC-M14, CCRC-M129, CCRC-M72
Linseed Mucilage RG-I	JIM3, CCRC-M40, CCRC-M161, CCRC-M164
Physcomitrella Pectin	CCRC-M98, CCRC-M94
RG-Ia	CCRC-M5, CCRC-M2
RG-Ib	JIM137, JIM101, CCRC-M61, CCRC-M30
RG-Ic	CCRC-M23, CCRC-M17, CCRC-M19, CCRC-M18, CCRC-M56, CCRC-M16
RG-I/ Arabinogalactan	CCRC-M60, CCRC-M41, CCRC-M80, CCRC-M79, CCRC-M44, CCRC-M33, CCRC-M32, CCRC-M13, CCRC-M42, CCRC-M24, CCRC-M12, CCRC-M7, CCRC-M77, CCRC-M25, CCRC-M9, CCRC-M128, CCRC-M126, CCRC-M134, CCRC-M125, CCRC-M123, CCRC-M122, CCRC-M121, CCRC-M112, CCRC-M21, JIM131, CCRC-M22, JIM132, JIM1, CCRC-M15, CCRC-M8, JIM16
Arabinogalactan-1	JIM93, JIM94, JIM11, MAC204, JIM20
Arabinogalactan-2	JIM14, JIM19, JIM12, CCRC-M133, CCRC-M107
Arabinogalactan-3	JIM4, CCRC-M31, JIM17, CCRC-M26, JIM15, JIM8, CCRC-M85, CCRC-M81, MAC266, PN 16.4B4
Arabinogalactan-4	MAC207, JIM133, JIM13, CCRC-M92, CCRC-M91, CCRC-M78
Unidentified	MAC265, CCRC-M97
